# Effects of CpG Oligodeoxynucleotide 1826 on transforming growth factor-beta 1 and radiation-induced pulmonary fibrosis in mice

**DOI:** 10.1186/s12950-016-0125-4

**Published:** 2016-05-17

**Authors:** Xuan Li, Guoxiong Xu, Tiankui Qiao, Sujuan Yuan, Xibing Zhuang, Jihong Zhang, Hui Bin Sun

**Affiliations:** Department of Radiation Oncology, Jinshan Hospital, Fudan University, Shanghai, China; Central Laboratory, Jinshan Hospital, Fudan University, Shanghai, China; Department of Radiation Oncology, Albert Einstein College of Medicine, Bronx, NY USA

**Keywords:** CpG Oligodeoxynucleotide1826, Radiation pulmonary fibrosis, TGF-β1, FoxP3

## Abstract

**Background:**

Cytosine-phosphate-guanine (CpG) oligodeoxyribonucleotides (ODNs) are synthetic DNA fragments containing unmethylated cytosine-guanine motifs with potential immune modulatory effects and have recently been suggested to enhance sensitivity to traditional therapies in lung cancer. This study aimed to examine the effects of CpG ODN1826 on transforming growth factor-beta 1(TGF-β1) and radiation-induced pulmonary fibrosis in mice.

**Methods:**

The radiation-induced pulmonary fibrosis mouse model was established by a single dose of 20 Gy, 6 MV X-rays exposure to the left lung. ICR mice were evenly randomized into four groups, comprising: a control group, a radiation group (RT group), a CpG group and a radiation combined with CpG ODN1826 group (RT + CpG group), with 40 mice in each group. CpG ODN1826 was intraperitoneally injected into mice at 1, 3, 5, 7 and 9 d post-irradiation. The mice were sacrificed at 1, 5, 15, 30 and 90 d post-irradiation. Paraffin sections of the radiated lung were subjected to H&E staining and Masson staining. The Ashcroft scale was used for quantitative histological analysis of fibrotic changes induced by irradiation. Concentrations of serum TGF-β1 were determined by ELISA, and concentrations of Hydroxyproline(Hyp) in the lung were determined with the alkaline hydrolysis method. Relative gene expression of FoxP3 was determined by real-time PCR.

**Results:**

The radiation-induced pulmonary fibrosis mouse model was successfully established. The serum concentrations of TGF -β1 of RT group were higher than those of the RT + CpG group (*t* = 5.212, 7.126, 7.972 and 3.785, *P* < 0.05). The Hyp in the lung of RT group was higher than that of RT + CpG group (*t* = 4.606, *P* < 0.05). The relative expressions of FoxP3 gene in the lung of the RT group were higher than those of RT + CpG group (*t* = 8.395, 5.099 and 6.147, *P* < 0.05).

**Conclusions:**

CpG ODN1826 could reduce the serum concentrations of TGF-β1 and the lung content of Hyp in radiation-induced pulmonary fibrosis, which might be related to the possibility that CpG ODN1826 can reduce expression of the FoxP3 gene.

## Background

Radiation therapy is one of the most important treatments for the chest tumors, but common complications from such treatments include radioactive lung injuries and dose-limiting side effects [1]. Radiation-induced pulmonary fibrosis is the main pathological process of late radiation induced lung injury [2]. TGF-β1, which plays an important role in the process of starting and developing pulmonary fibrosis, has been acknowledged as the most important cytokine to reflect pulmonary fibrosis severity [3]. The FoxP3 gene is one of the important genes regulating the secretion of TGF-β1, and FoxP3 gene expression can reduce the secretion of TGF-β1. CpG oligodeoxynucleotides (ODNs) are synthetic DNA sequences containing unmethylated cytosine-guanine motifs, which were identified by activating TLR9 in antigen-presenting cells and B cells, and CpG ODNs can activate the active immune cells to produce a variety of cytokines which enhance the body’s specific and nonspecific immune effect and prevent a potential microbial infection [4]. CpG can improve the microenvironment in some malignant tumors, such as lung cancer and liver cancer, and reduce the TGF-β1 concentration in the microenvironment [5, 6]. Therefore, CpG might also reduce the TGF-β1 concentration in radiation-induced pulmonary fibrosis disease. In this study, we observed the effects of CpG on radiation-induced pulmonary fibrosis, detected the effects of CpG on the serum TGF-β1 and preliminary explored if this effect was related to the CpG FoxP3 gene.

## Methods

### Experimental animals and reagents

One hundred and sixty ICR female mice were provided by the Shanghai Experimental Animal Center and maintained in a specific pathogen-free grade animal room until 6–8 weeks of age and weighing 18–22 g. The study was approved by the ethics committee of Jinshan Hospital of Fudan University. CpG ODN1826 was purchased from Shanghai Biological Engineering Technology and Service Limited Company (Shanghai, China). CpG ODN1826 was completely phosphorothioate-modified and purified with PAGE gel. The sequence of CpG ODN1826 was: 5′-TCC ATG ACG TTC CTG ACG TT-3′. CpG ODN1826 was diluted in PBS to a concentration of 0.1 mg/ml. The real time PCR primer sequences for FoxP3 were: upstream primer: 5′-TTCACCTATGCCACCCTTATCC-3′, downstream primer: 5′-GCGAAACTCAAATTCATCTACGG-3′; GAPDH: upstream primer: 5′-GCCTTCCGTGTTCCTACC-3′, downstream primer: 5′- AGAGTGGGAGTTGCTGTTG-3′. Mouse serum TGF-β1 ELISA kit was provided by R&D Company from USA. Masson Trichromatic staining kit and hydroxyproline (Hyp) kit were provided by Nanjing Kaiji Biological Science and Technology Limited Company (Nanjing, China). ReverTra Ace®qPCR RT kit was provided by TOYOBO Company from Japan, Trizol from Invitrogen Company from USA and RevertAid TMFirst strand cDNA Synthesis kit from Fermentas Company from Canada. A linear accelerator was used (Precise 5839, Elekta, Stockholm, Sweden).

### Experimental groups

One hundred and sixty ICR mice were evenly randomized into four groups: (1) Control group: intraperitoneal injection of 0.2 ml saline. (2) RT group: intraperitoneal injection of the same saline with single left lung 20 Gy of 6 MV X-ray. (3) CpG group: intraperitoneal injection of 0.1 mg/ml concentration CpG ODN1826 solution 0.2 ml. (4) RT + CpG group: intraperitoneal injection of the same CpG ODN1826, with single left lung 20 Gy of 6 MV X-ray. The first injections were performed 12 h after radiation, which was defined as day 1. The saline and CpG ODN1826 groups were all injected on day 1, 3, 5, 7 and 9. Six mice of each group were sacrificed on day 1, 5, 15, 30 and eight mice on day 90.

### Radiation-induced pulmonary fibrosis mouse model

Before irradiation, mice were anesthetized by injecting intraperitoneal 4 μl/g body mass of 10 % chloral hydrate. The mice were immobilized and shielded under a home-made device. After accurate positioning of the irradiation area of mice with the simulator, a single dose of 20Gy of 6 MV X-rays was delivered to a 2 cm × 2 cm area in the left lung at a rate of 2.0 Gy/min. Non-irradiated mice underwent the same procedure but were not exposed to radiation.

### Histologic analysis and pulmonary fibrosis scoring

Lung tissues were dissected at different time points and fixed in formalin. The tissues were then embedded in paraffin and sectioned. After H&E staining or Masson staining, pulmonary fibrosis was observed under an optical microscope. The Ashcroft scale was used for the quantitative histologic analysis of fibrotic changes induced by irradiation. Pulmonary fibrosis was scored double blinded under the optical microscope. The pulmonary fibrosis was scored as follows: 0, normal; 1, a little fibrosis of alveolar bronchiole wall; 2–3, moderate fibrosis and no structural changes of alveolar wall; 4–5, alveolar structure changes and fibers; 6–7, serious alveolar structure changes and massive fibrosis; 8, totally fibrosis [7]. Each sample was scored with 9 horizons randomly, and the average score of each group was calculated.

### Detecting the serum TGF-β1 and Hyp concentrations in the radiated mouse lung tissues

Mouse blood samples were collected under institute approved protocols and incubated at room temperature for 30 min to allow the blood to clot. The blood samples were then centrifuged at 3000 rpm for 15 min. Sera were then collected and diluted 60-fold to test TGF-β1 concentrations by ELISA. Hyp concentrations in the radiated lung tissues were determined with the alkaline hydrolysis method. Briefly, freshly irradiated lung tissue was hydrolyzed with an accurate scale under the PH at 6.0–6.8. The supernatant was collected after centrifugation. The absorbance of each sample was measured with a light spectrophotometer under 550 nm and Hyp concentration of each sample was calculated.

### Expression of FoxP3 gene in the irradiated mouse lungs

Total RNA samples were isolated from flash-frozen left lung tissues of the mice from each group with Trizol by following the manufacturer’s instructions. First strand cDNA was synthesized with the reverse transcription kit. Relative mRNA expression of the FoxP3 gene was assayed by quantitative real time PCR with gene specific primers with GAPDH as internal control.

### Statistical analysis

Statistical analysis was performed by SPSS21.0 (IBM). Data were expressed as means ± standard deviation (SD). Single factor analysis of variance test was used to test the differences between groups. The multiple comparisons were evaluated by the Bonferroni method. The non-parametric statistic test (Wald-Wolfowitz test) was used to test for differences between RT group and RT + CpG group on pulmonary fibrosis scores. Differences resulting in *P* < 0.05 were considered to be statistically significant.

## Results

### CpG reduced radiation-induced pulmonary fibrosis

**Fig. 1:**
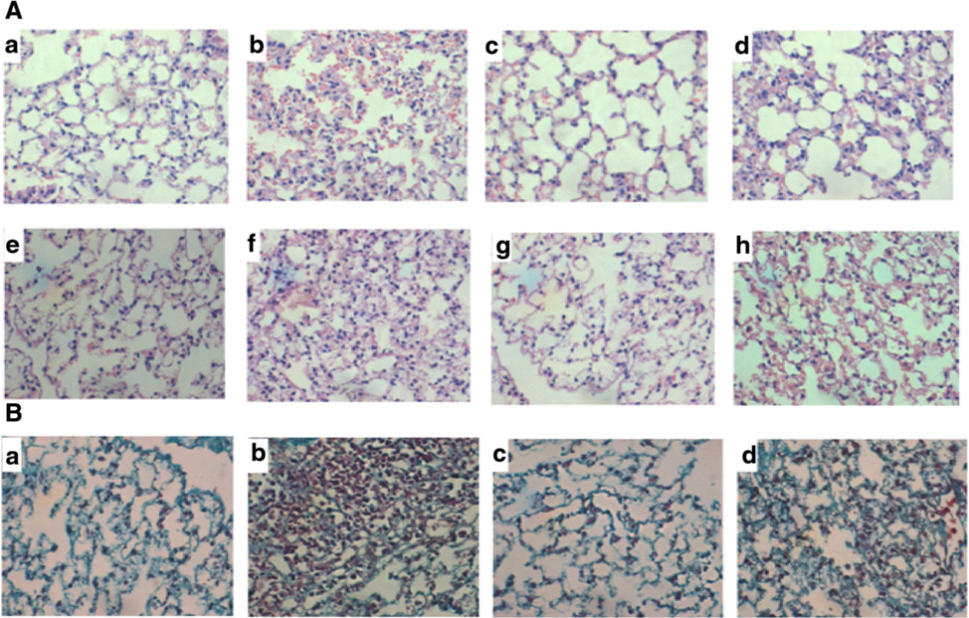
**a** Left lung tissue paraffin section with HE staining (×200); *a*: Control group days 30 after irradiation; *b*: RT group days 30 after irradiation; *c*: CpG group days 30 after irradiation; *d*: RT + CpG group days 30 after irradiation; *e*: Control group days 90 after irradiation; *f*: RT group days 90 after irradiation; *g*: CpG group days 90 after irradiation; *h*: RT + CpG group days 90 after irradiation. **b** Left lung tissue section with Masson staining (×200); *a*: Control group days 90 after irradiation; *b*: RT group days 90 after irradiation; *c*: CpG group days 90 after irradiation; *d*: RT + CpG group days 90 after irradiation. Abbreviations: CpG, cytosine-guanine; RT, irradiation

**Fig. 2:**
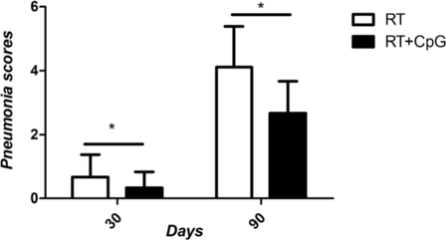
The scores of pulmonary fibrosis on day 30 and 90. Notes: ^*^ *P* < 0.05, RT versus RT + CpG on days 30 and 90. Abbreviations: CpG, cytosine-guanine; RT, irradiation

We observed the structural changes of bronchi, alveoli and alveolar interval organization under the optical microscope. Figure 1a a e c g and Fig. 1b a c showed the bronchi, alveoli and alveolar interval structure of the pseudo irradiation groups were normal, with no chronic inflammation change and pulmonary fibrosis. Therefore, the pulmonary fibrosis scores of Control and CpG groups were zero. Pulmonary fibrosis of RT and RT + CpG groups were not observed on day 5 and 15. Figure 1a b showed part of the fiber cells and collagen fiber hyperplasia, a lot of neutrophils and mononuclear macrophage infiltration, and inflammatory cells gathered around secondary bronchi and blood vessels in the RT group on day 30. Figure 1a d showed the RT + CpG group exhibited less pulmonary hemorrhage, the fiber cells and collagen fiber hyperplasia, neutrophils and mononuclear macrophage infiltration than the RT group on day 30. Figure 1a f and Fig. 1b b showed the red blood cells and inflammatory cells infiltration of the RT group on day 90 were reduced, but part of the alveolar cavity disappeared, alveolar wall fractured and a large number of fiber cells proliferated and collagen fibers were deposited in the alveolar interval. Figure 1a h and Fig. 1b d showed basic alveolar structure in the RT + CpG group was normal, with regional focal fibrosis nodules, and less fiber cell proliferation and collagen deposition observed between alveolus on day 90. The results in Fig. 2 showed the pulmonary fibrosis score of RT group was higher than that of RT + CpG group on day 30 and 90. And the difference was statistically significant on day 90 (*P* < 0.05). This data indicates CpG may play a protective role in radiation-induced pulmonary fibrosis.

### CpG ODN1826 reduced serum TGF-β1 concentrations in irradiated mice

TGF-β1 plays a vital role in the initiation and progression of pulmonary fibrosis, and has been acknowledged as the most important cytokine to reflect the severity of pulmonary fibrosis [3]. The results in Table 1 showed serum TGF-β1 concentrations in mice increased after irradiation, and the highest point was detected on day 30. The serum TGF-β1 concentrations of the RT + CpG group were lower than those of the RT group on day 5, 15, 30 and 90 (*P* < 0.05). From the results, we found that CpG could reduce the serum TGF-β1 concentrations in radiation-induced pulmonary fibrosis mice.

**Table 1:** The serum TGF-β1 at different times (days) (ng/ml, $\overline{x}+s$)

Group	Mice(n)	1 d	5 d	15 d	30 d	90 d
Control	6	107.511 ± 10.032	103.027 ± 11.797	117.598 ± 16.270	108.595 ± 17.082	123.615 ± 17.491
RT	6	137.800 ± 9.694^a^	171.247 ± 13.449^ac^	203.633 ± 9.775^ac^	237.516 ± 11.589^ac^	174.161 ± 16.690^ac^
CpG RT + CpG	6 8	116.985 ± 17.541 120.275 ± 21.534	122.065 ± 22.832 138.600 ± 7.388^ab^	105.213 ± 19.539 157.809 ± 12.353^abc^	112.231 ± 14.892 180.615 ± 13.091^abc^	102.731 ± 16.177 146.649 ± 12.004^bc^

Notes: *F* = 4.959, *P* < 0.05 on days 1; *F* = 23.1, *P* < 0.05 on days 5; *F* = 51.743, *P* < 0.05 on days 15; *F* = 114.643, *P* < 0.05 on days 30; *F* = 30.874, *P* < 0.05 on days 90; ^a^ *P* < 0.05 versus control at t = 5.318, 9.432, 11.103, 15.298, 5.913, 6.260, 4.822 and 8.197; ^b^ *P* < 0.05 versus RT at t = 5.212, 7.126, 7.972 and 3.785; ^c^ *P* < 0.05 versus CpG at t = 4.546, 11.034, 16.263, 8.692, 5.573, 8.448 and 6.167

*Abbreviations: CpG* cytosine-guanine, *RT* irradiation, *n* number, *d* days

### CpG ODN1826 decreased concentrations of Hyp in the irradiated mouse lung

During the progression of radiation-induced pulmonary fibrosis, the main sedimentary composition are collagen fibers [8]. Hyp is characteristic of collagen fiber, and it is commonly used to reflect the degree of radiation-induced pulmonary fibrosis [9]. From the pathological sections, we observed that the radiation-induced pulmonary fibrosis was most severe on day 90 after irradiation. We therefore measured Hyp concentrations in the radiated lung tissues in mice on day 90 to quantify the degree of radiation-induced pulmonary fibrosis. The results in Table 2 showed the radiated lung tissues concentrations of Hyp in mice increased on day 90. The Hyp concentration in radiated lung tissue of the RT + CpG group was lower than that of the RT group (*P* < 0.05). This result was consistent with the results observed in the pathological sections. These data together with those obtained from the pathological sections suggest that CpG could reduce sedimentary collagen fiber in irradiated lungs, and alleviate radiation-induced pulmonary fibrosis.

**Table 2:** The lung tissue concentrations of Hyp on day 90 (μg/mg, $\overline{x}+s$)

Group	Mice(n)	90 d
Control	8	1.137 ± 0.087
RT	8	1.733 ± 0.196^ac^
CpG RT + CpG	8 8	1.090 ± 0.122 1.388 ± 0.080^abc^

Notes: *F* = 57.470, *P* < 0.05; ^a^ *P* < 0.05 versus control at t = 7.847 and 6.003; ^b^ *P* < 0.05 versus RT at t = 4.606; ^c^ *P* < 0.05 versus CpG at t = 7.867 and 5.778

*Abbreviations: CpG* cytosine-guanine, *RT* irradiation, *n* number, *d* days

### CpG treatment down regulates FoxP3 expression in irradiated lung

The FoxP3 gene is one of the important genes in regulating the secretion of TGF-β1, and studies have confirmed that FoxP3 gene expression can reduce the secretion of TGF-β1 [10–12]. The relative expressions of FoxP3 was analyzed by real-time PCR. The results in Table 3 showed that the relative expression quantity of FoxP3 in irradiated lung increased after radiation. The relative expression quantity of FoxP3 in the RT + CpG group was lower than that in the RT group (*P* < 0.05). The results demonstrate CpG treatment down-regulates FoxP3 expression in irradiated lungs, and this may be the reason that CpG ODN1826 could reduce the serum concentration of TGF-β1 of radiation-induced pulmonary fibrosis mouse.

**Table 3:** Relative expressions of FoxP3 at different times (days) (2^-△CT△CT^, $\overline{x}+s$)

Group	Mice(n)	5 d	15 d	30 d
Control	6	1.000 ± 0.181	1.000 ± 0.105	1.000 ± 0.135
RT	6	2.322 ± 0.225^ac^	2.498 ± 0.389^ac^	2.123 ± 0.266^ac^
CpG RT + CpG	6 6	0.452 ± 0.081 1.498 ± 0.086^abc^	0.458 ± 0.024 1.617 ± 0.167^abc^	0.408 ± 0.104 1.364 ± 0.144^abc^

Notes: *F* = 155.5, *P* < 0.05 on days 5; *F* = 96.553, *P* < 0.05 on days15; *F* = 102.525, *P* < 0.05 on days 30; ^a^ *P* < 0.05 versus control at t = 11.224, 9.115, 9.214, 6.072, 7.661, and 4.521; ^b^ *P* < 0.05 versus RT at t = 8.395, 5.099 and 6.147; ^c^ *P* < 0.05 versus control at t = 19.182, 12.833, 14.709, 21.585, 16.807 and 13.228

*Abbreviations: CpG* cytosine-guanine, *RT* irradiation, *n* number, *d* days

## Discussion and conclusions

CpG oligodeoxynucleotides (ODNs) are synthetic DNA sequences containing unmethylated cytosine–guanine motifs, which activate TLR9, and CpG ODNs can activate many kinds of immune cells [4]. CpG ODNs can cause the differentiation of B cells, stimulate B cells to secrete cytokines IL-6 and IL-10, and prevent apoptosis of B cells [13]. In cooperation with IL-12, CpG ODNs can activate NK cells directly to secrete IFN-γ, which in turn, can further promote the activation of monocytes, macrophages and dendritic cells [14]. At present, the research of the CpG as immune adjuvant is thorough. In the CpG ODN auxiliary hepatitis b vaccine clinical trials, researchers found that CpG ODN can effectively activate the body’s immune system to produce the cellular and humoral immune response with the hepatitis b vaccine, and found no obvious toxic effects [15, 16]. It has been proven that CpG ODNs can treat infectious diseases, cancer and allergic diseases [17–19]. For example, a study found that protamine nanoparticles with CpG-oligodeoxynucleotide prevent an allergen-induced Th2-response in BALB/c mice [20]. Our previous experiments gave mice ODN1826 through intraperitoneal injection on day 1, 3, 5, 7 and 9, and found that CpG ODN1826 has obvious radio-sensitization effects to lung cancer, and no obvious cytotoxicity was found [21]. Radiation-induced pulmonary fibrosis is the main pathological process of the late radiation induced lung injury [2]. In the early damage, inflammatory mediators secretion increased continuously, causing alveolar exudates to increase, pulmonary interstitial hyperemia edema and inflammatory reaction cells infiltration. In the late damage, an exception repairing and tissue remodeling occurred, muscle fibroblasts increased, which resulted collagen deposition and fibrosis [8, 22].

Recent studies suggest that the pathological morphological changes of radiation-induced lung injury mainly include pulmonary interstitial hyperemia and edema, an increase in alveolar exudates, inflammatory cells infiltration, late fiber connective tissue hyperplasia and alveolar atrophy [23, 24]. The H&E staining and Masson staining of paraffin sections showed the radiation-induced pulmonary fibrosis mouse model was clearly established in this study. CpG ODN1826 could reduce fiber cell proliferation and collagen deposition. Hyp accounts for 13.4 % of collagen and is often used to reflect pulmonary fibrosis [9]. Our study found that CpG ODN1826 could reduce the Hyp content of the exposed lung. From these results, we posit that CpG ODN1826 might ameliorate pulmonary fibrosis caused by irradiation.

TGF-β is the growth regulating factor for the epithelial origin cells in human, and has extensive biological effects in cell growth, differentiation, extracellular matrix deposition and the immune reaction of the body [25]. TGF-β1, one of the subtypes of TGF-β, has been acknowledged as the most important cytokine to result the fibrosis in late injury, which can not only induce the fibroblast around the bronchioles branch and vascular into myofibroblasts, but also induce the normal lung epithelial cells into myofibroblasts, and thus play an important role in the process of fibrosis [26, 27]. CpG ODNs may affect the secretion of various cytokines. However, the effect of CpG ODN1826 to TGF-β1 and radiation-induced pulmonary fibrosis is unknown. Our study found that the serum TGF-β1 concentrations in mice increased after irradiation, and the highest point we detected was on day 30. However, the serum TGF-β1 concentration of the RT + CpG group was lower than that of the RT group, suggesting CpG ODN1826 could reduce the serum concentration of TGF-β1 after irradiation. CD4+/CD25+ Treg, which has the function of regulating the immune system, is a T cell subgroup, and is an important factor for the secretion of TGF-β1 [28]. FoxP3 is the most specific symbol of CD4+/CD25+ Treg, and the key regulatory gene in the development and functions to maintain CD4+/CD25+ Treg [29]. One study has found that CpG ODNs could inhibit the expression of the FoxP3 gene, and reduce the proportion of CD4+/CD25+ Treg, and thus down-regulate the serum TGF-β concentration in lung cancers [5]. Our study found that the expression of the FoxP3 in mice increased after irradiation, but the expression of the FoxP3 in RT + CpG group was lower than that of irradiation group. Therefore, CpG ODN1826 could reduce the serum concentration of TGF-β1 of radiation-induced pulmonary fibrosis mouse, which might be due to its effect on reduction of FoxP3 gene expression. Compared with the control group, FoxP3 gene was downregulated in the CpG group. However, as shown in Table 1, no significant difference was observed in the TGF-β1 expression of CpG group and the control group. One of the possible explanations is that CpG, as an immunopotentiator, exerts its effect on balancing T1/T2 immune cells. The mechanisms of how Foxp3 regulates TGF-β1 expression are still not fully understood. Peng et al. found that CD4 + CD25 + FoxP3+ Tregs attenuate TGF-β1 induced lung fibrosis and fibrocyte accumulation in part via suppression of FGF-9 [11]. Sumitomo et al. found that transcription factor early growth response 3 is a key factor associated with TGF-β1 expression regulated by FoXp3 [12]. Other than downregulation of FoxP3 gene expression, CpG might balance the secretion of TGF-β1 via some other mechanism. In addition, there are also many other factors may affect TGF-β1 expression. For example, Molteni et al. found that angiotensin had an important regulatory role on TGF-β1, and it promoted the secretion of TGF-β1 in the process of radiation-induced lung injury [30]. Jain found that plasma endothelin-1 could stimulate the secretion of TGF-β1 in the study of idiopathic pulmonary fibrosis [31]. We will therefore take further steps to elucidate the mechanism in our future studies. In conclusion, our study found that CpG ODN1826 could partly prevent radiation-induced pulmonary fibrosis, and reduce the serum concentration of TGF-β1 of radiation-induced pulmonary fibrosis, which might be related to the reduced expression of the FoxP3 gene. This finding indicatives a significant potential for the clinical application of CpG ODNs in treatment of the radiation-induced pulmonary fibrosis.

## Abbreviations

CpG ODN1826: CpG oligodeoxynucleotide 1826
Hyp: hydroxyproline
TGF-β1: transforming growth factor-beta 1
